# How to Promote Skin Repair? In-Depth Look at Pharmaceutical and Cosmetic Strategies

**DOI:** 10.3390/ph16040573

**Published:** 2023-04-11

**Authors:** Ana Torres, Liliana Rego, Márcia S. Martins, Marta S. Ferreira, Maria T. Cruz, Emília Sousa, Isabel F. Almeida

**Affiliations:** 1 UCIBIO—Applied Molecular Biosciences Unit, MedTech, Laboratory of Pharmaceutical Technology, Department of Drug Sciences, Faculty of Pharmacy, University of Porto, 4050-313 Porto, Portugal; 2 Associate Laboratory i4HB, Institute for Health and Bioeconomy, Faculty of Pharmacy, University of Porto, 4050-313 Porto, Portugal; 3 Laboratory of Organic and Pharmaceutical Chemistry, Department of Chemical Sciences, Faculty of Pharmacy, University of Porto, 4050-313 Porto, Portugal; 4 CIIMAR—Interdisciplinary Centre of Marine and Environmental Research, Avenida General Norton de Matos, S/N, 4450-208 Matosinhos, Portugal; 5 Faculty of Pharmacy, University of Coimbra, 3004-531 Coimbra, Portugal; 6 Center for Neuroscience and Cell Biology, 3004-504 Coimbra, Portugal

**Keywords:** skin repair, wound healing, trends, scientific evidence, epidermal barrier, metal oxides and salts

## Abstract

Skin repair encompasses epidermal barrier repair and wound healing which involves multiple cellular and molecular stages. Therefore, many skin repair strategies have been proposed. In order to characterize the usage frequency of skin repair ingredients in cosmetics, medicines, and medical devices, commercialized in Portuguese pharmacies and parapharmacies, a comprehensive analysis of the products’ composition was performed. A total of 120 cosmetic products, collected from national pharmacies online platforms, 21 topical medicines, and 46 medical devices, collected from INFARMED database, were included in the study, revealing the top 10 most used skin repair ingredients in these categories. A critical review regarding the effectiveness of the top ingredients was performed and an in-depth analysis focused on the top three skin repair ingredients pursued. Results demonstrated that top three most used cosmetic ingredients were metal salts and oxides (78.3%), vitamin E and its derivatives (54.2%), and *Centella asiatica* (L.) Urb. extract and actives (35.8%). Regarding medicines, metal salts and oxides were also the most used (47.4%) followed by vitamin B5 and derivatives (23.8%), and vitamin A and derivatives (26.3%). Silicones and derivatives were the most common skin repair ingredients in medical devices (33%), followed by petrolatum and derivatives (22%) and alginate (15%). This work provides an overview of the most used skin repair ingredients, highlighting their different mechanisms of action, aiming to provide an up-to-date tool to support health professionals’ decisions.

## 1. Introduction

*Stratum corneum* (SC) is the outermost skin layer and is constituted by corneocytes embedded in a multilamellar lipid matrix containing ceramides, cholesterol, and free fatty acids (FFA) in equimolar ratio, maintained by regulatory fine-tuning mechanisms [1]. The integrity of the SC is the primary determinant of the skin’s barrier function. As the skin is exposed to multiple exogenous factors that may lead to epidermal barrier (EB) impairment, the SC is continuously active in maintaining a functional physiologic state by resorting to a variety of self-repair mechanisms. Once transepidermal water loss (TEWL) is increased beyond the normal homeostatic level, multiple self-repair mechanisms are induced within the SC and at the granular zone transition layer of the epidermis. Initially, an inflammatory cascade triggers the release of tumor necrosis factor (TNF), interleukin (IL)-1, and IL-6, promoting keratinocyte hyperproliferation. This increased epidermal thickness aims to counter the excessive TEWL. Simultaneously, an increased synthesis of SC lipids is evoked, such as cholesterol and ceramides, and constituents of the natural moisturizing factor (NMF), namely urea, pyrrolidone carboxylic acid, lactic acid, urocanic acid, and various amino acids, replenishing the SC structure [2]. Some skin disease states, such as atopic dermatitis, innate xerosis, ichthyosis, psoriasis, diabetes, and increased age, exhibit inherent SC impairment, predisposing to an increased TEWL [3,4]. Therefore, if these self-repair mechanisms fail, or do not meet the needs of the skin barrier, clinical signs are triggered, such as scaling and flaking, loss of elasticity, micro and macro fissures, and hyperkeratosis [3]. In addition to the epidermal barrier, skin injury can also affect the dermis. Wounds result from the disruption of the both epidermis, dermis, and deep subcutaneous fat and blood vessels (full thickness wound), the epidermis and superficial dermis (partial thickness wound), or only the epidermis (superficial wound) due to thermal or physical damage, thus compromising the cohesiveness of the epithelium [5,6,7]. Wound healing consists of a dynamic, interactive, and orchestrated process, involving four phases—hemostasis, inflammation, proliferation, and remodeling—that partially overlap with each other [8,9]. Depending on the extent of the lesion this process may not always involve all phases. Immediately after a trauma, if blood vessels are affected, platelet activation triggers fibrin production and subsequent clot formation, inducing a hemostatic effect [10]. An inflammatory response is readily initiated and, as a result of platelet degranulation and release of both chemotactic signals and bacterial degradation products, the immune innate system is activated, and neutrophils and monocytes are recruited to the wounded tissue [10,11]. Monocytes differentiate into macrophages, inducing an active defense response against foreign bodies, as well as secretion of chemokines and growth factors (GFs) [10]. The proliferation phase takes place within two to three days after the wound generation, when keratinocytes induce re-epithelialization. Endothelial cells promote angiogenesis and fibroblasts begin to form new extracellular matrix [12]. Lastly, in the remodeling phase, the previous inflammatory processes are downregulated and substituted by events aiming to reorganize the connective tissue, initiating the contractile response [9]. Any disruption of these physiological processes can impair wound healing or lead to chronic wounds, potentiating the formation of hypertrophic scars, keloids, or excessive scar tissue [9].

The different mechanisms by which cosmetic products, medicines, and medical devices promote skin repair are under different regulations. According to Regulation (EC) 1223/2009, a cosmetic product is “(…) intended to come into contact exclusively with the external part of the body with a view to cleaning it, perfuming it, changing its appearance, protecting it, keeping it in good condition or correcting body odours” [13]. On the other hand, according to Directive 2001/83/EC and Decree Law No. 176/2006, medicines include substances or compositions of substances that possess properties for treating or preventing disease and its symptoms with a view to making a medical diagnosis or to restore, correct, or modify its functions [14,15]. Medical devices, which are under the regulation (EU) 2017/745, present common purposes to those of medicinal products, such as preventing, diagnosing, or treating human disease. “Medical device” is any instrument, apparatus, appliance, software, implant, reagent, material, or other article intended by the manufacturer to be used in human beings for medical purposes. This definition includes devices that are used for diagnosis, prevention, monitoring, treatment, or alleviation of disease or injury in humans [16]. Contrary to medicines, their mechanisms of action cannot be translated into pharmacological, metabolic, or immunological actions, as their effect is achieved by providing physical protection [16]. Medical devices are divided into different classes: class I, less critical (low risk) devices, most of the non-active or non-invasive ones that can either be sterile or perform a measurement function; class II, medium risk active or non-active devices, either invasive or non-invasive, that interact with the body in a non-dangerous (Class IIa) or in a dangerous (Class IIb) way; class III, high-risk devices, mostly implantable, containing drugs or animal derivatives and aiming vital organs functions [17]. Wound healing medical devices could be integrated into all the classes depending on the technology, type of wound, and depth of the damaged tissue [17,18]. While the action of cosmetic products is restricted to the epidermis, medicines, and medical devices can have a deeper action, either at dermal or systemic levels. Therefore, it is foreseeable that the ingredients incorporated in these different formulations, or the concentration in which they are found, are not the same. Substances with an active role in the inflammatory phase or in the regeneration phase are often incorporated into skincare formulations. They can act by neutralizing free radical species or reducing pro-inflammatory mediators, or in the regeneration phase, either by improving collagen synthesis or inhibiting its degradation [19,20]. Both topical drugs and cosmetic products can impact the damaged skin barrier namely through the improvement of skin hydration [21]. This moisturizer effect is provided by ingredients that boost the synthesis of skin structural lipids, restoring directly the skin barrier (i.e., vitamin A), or by hygroscopic substances (i.e., dexpanthenol) that bind and retain water to the SC, therefore reducing the TEWL [22]. Emollient ingredients (i.e., petrolatum derivatives) also promote hydration by directly replacing essential fatty acids that might be lacking in skin [23]. Proper skin hydration provides flexibility, protecting it from damage and promoting the desquamation process, enabling the activation of hydrolytic enzymes [24]. Wound healing medical devices traditionally used in clinical practice (gauze, sterilized absorbent cotton, and bandages) can only offer physical protection, revealing limited benefits directly on the wound healing process and prevention of infection [18]. Therefore, modern dressings such as hydrogels, hydrocolloids, alginates, foams, and films are being developed to maintain the wound microenvironment, providing a relatively constant local temperature and humidity, thus facilitating re-epithelialization and preventing further skin damage, while avoiding contact with external bacteria [18,25].

The aim of this work is to provide the reader with an up-to-date overview of the most used skin repair ingredients in cosmetic products, medicines, and medical devices currently commercialized, as well as the mechanisms of action behind the skin regenerating effectiveness. 

## 2. Results

### 2.1. Overview of the Use of Wound Repair Ingredients in Cosmetic Products, Medicines, and Medical Devices

#### 2.1.1. Cosmetic Products

**Figure 1:**
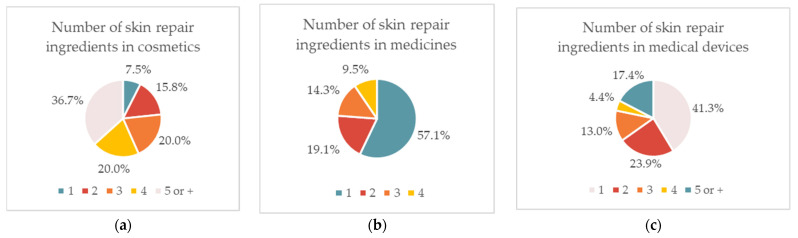
The prevalence of wound repair ingredients or active substances in cosmetic products (**a**), medicines (**b**), and medical devices (**c**).

The presence of skin repair ingredients was analyzed in 120 cosmetic products, 21 topical medicines, and 46 medical devices, commercialized in Portugal in 2022. The analysis of the respective labels revealed that within cosmetic products with skin repair ingredients, the highest incidence (36.7%) presents four or more skin repair ingredients, whereas only a small percentage (7.5%) contained only one ingredient with the same purpose (Figure 1a). On the contrary, in topical medicines, most of the labels analyzed displayed only one skin repair ingredient in their composition (57.1%) (Figure 1b), as was also observed for medical devices (41.3%) (Figure 1c).

**Figure 2:**
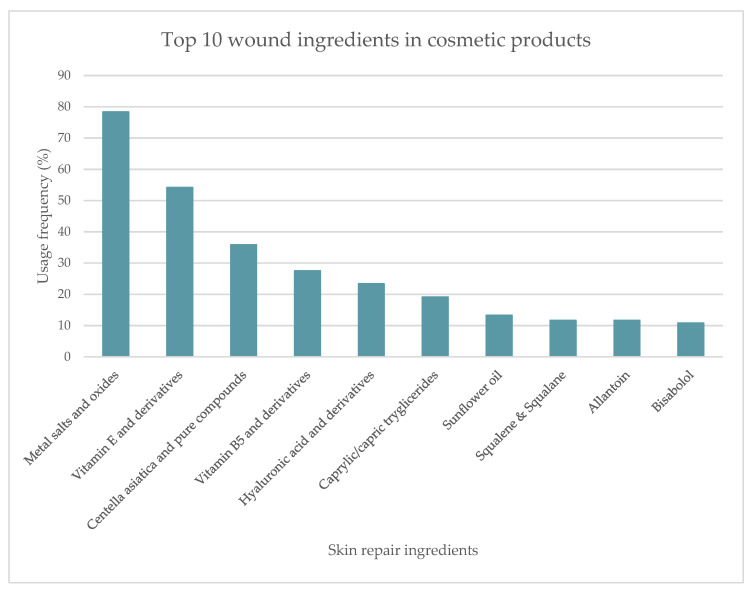
Usage frequency, in percentage, of the top 10 skin repair ingredients present in cosmetic products.

Among the 120 cosmetic products, a total of 64 ingredients with the ability to regenerate skin were identified. A preliminary analysis was performed, and the top 10 most used skin repair ingredients were listed (Figure 2). Metal salts and oxides were the most used skin repair ingredients (78.3%), followed by vitamin E and its derivatives, used in 54.2% of the analyzed products. *Centella asiatica* (L.) Urb. extract and its bioactives were the third most used ingredients (35.8%). The following ingredients, vitamin B5 and its derivatives (pantothenic acid, dexpanthenol, and panthenol), hyaluronic acid and salt forms, caprylic/capric triglycerides, sunflower oil, squalene and squalane, allantoin and bisabolol, showed a usage frequency between 10 to 30%.

**Figure 3:**
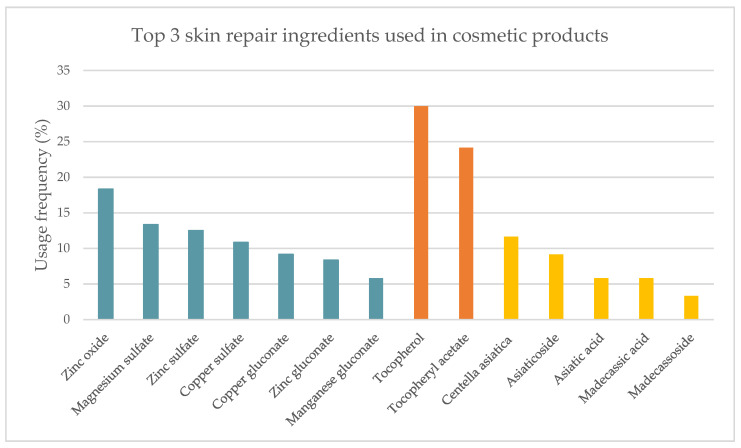
Usage frequency of the top 3 skin repair ingredients in cosmetics extended: metal salts and oxides (blue), vitamin E and derivatives (orange) and *C. asiatica* and pure compounds (yellow).

A detailed analysis of the three most used skin repair ingredients in cosmetic products was performed (Figure 3). The high prevalence of metal salts and oxides, found in 94 of the 120 analyzed cosmetic products, results essentially from the diversity of ingredients belonging to this category. Zinc oxide is the most widely used metal oxide in the studied cosmetics (18.3%). Magnesium, zinc, and copper sulfates are present in slightly lower percentages (13.3%, 12.5%, and 10.8%, respectively). Copper (9.2%), zinc (8.3%), and manganese gluconates (5.8%) complete the list of ingredients included in this group (Figure 3–blue). Vitamin E, also known as tocopherol, and its derivative, tocopheryl acetate, were the second most prevalent skin repair ingredients, with a usage frequency of 30.0% and 24.2%, respectively. *C. asiatica*, along with its bioactives, occupies the last place in the top three (11.7%). In addition to the use of *C. asiatica* as the whole extract, in some formulations, the bioactives are singly used: asiaticoside (9.2%), asiatic acid (5.8%), madecassic acid (5.8%), and madecassoside (3.3%) as illustrated in Figure 3.

#### 2.1.2. Topical Medicines

**Figure 4:**
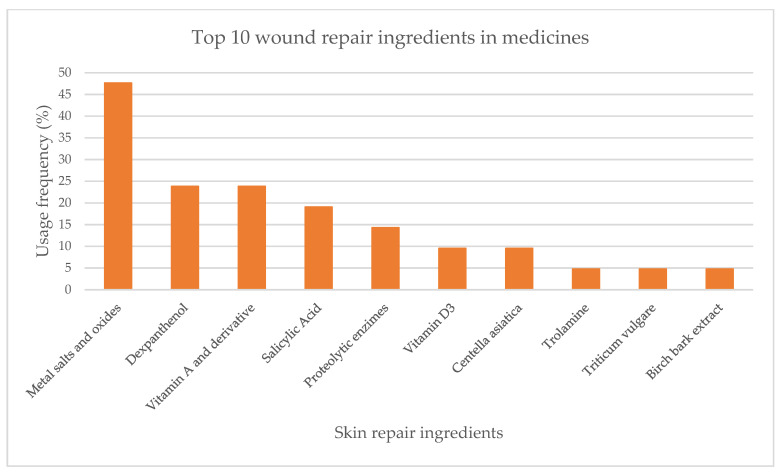
Usage frequency, in percentage, of the top 9 skin repair ingredients found in medicines.

From the analysis of labels and summary of products characteristics (SmPC) of 21 medicines, 10 active substances with skin repair function were identified. Metal salts and oxides were the most used skin repair ingredients (47.6%). Vitamin B5 and its derivative dexpanthenol (23.8%), along with vitamin A and its derivative (23.8%) were the following most used (Figure 4). Salicylic acid, proteolytic enzymes, vitamin D3, *C. asiatica*, trolamine, *Triticum vulgare* Vill. and birch bark extract presented usage frequencies below 20%.

**Figure 5:**
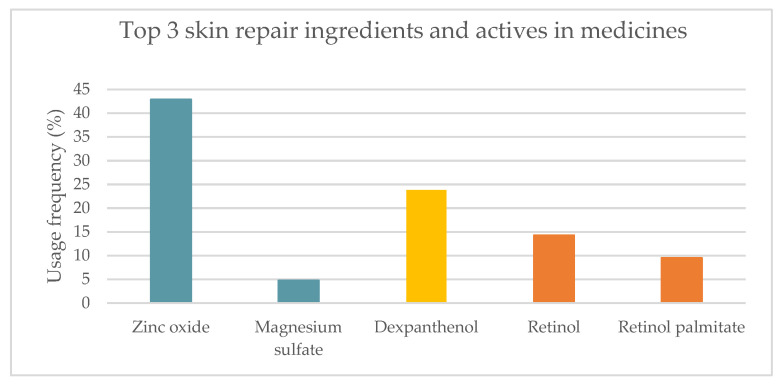
Usage frequency of the top 3 wound repair ingredients and actives in medicines extended: metal salts and oxides (blue), vitamin B5 derivative (yellow), vitamin A and derivative (orange).

The top three most used skin repair ingredients were identified and spanned (Figure 5). In accordance with what was observed in cosmetics, zinc oxide was the most used skin repair ingredient (42.9%). Magnesium sulfate was also included in the metal salts and oxides category, with a much lower usage frequency (4.8%). Only one vitamin B5 derivative, dexpanthenol, with a usage frequency of 23.8%, was present in the medicines analyzed, in contrast to what was observed in cosmetic products. Vitamin A, also known as retinol, (14.3%), along with its derivative, retinyl palmitate (9.5%), were the third most used skin regenerating ingredients in topical medicines.

#### 2.1.3. Medical Devices

**Figure 6:**
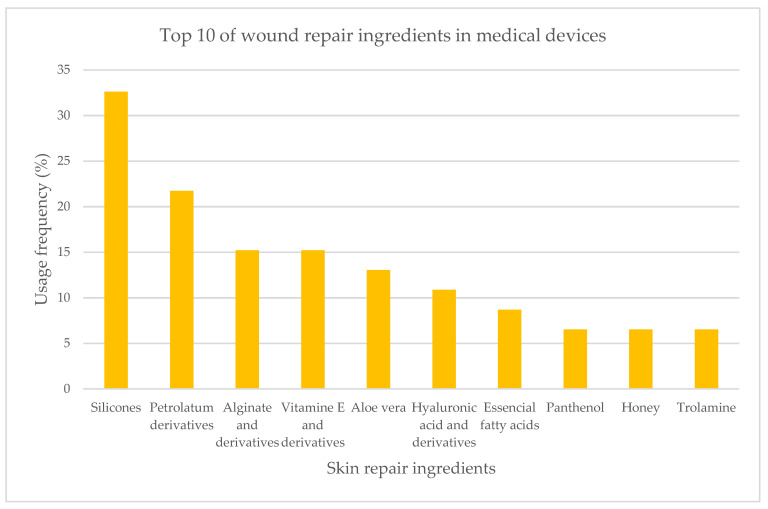
Usage frequency, in percentage, of the top 10 skin repair ingredients present in medical devices.

The analysis of 46 medical device labels and information leaflets revealed the top 10 most used ingredients in skin repair devices (Figure 6). More than 40 different active ingredients were identified, and silicones were revealed to be the preferred skin repair ingredients applied in semi-solid formulations (32.6%). Petrolatum derivatives (21.7%) followed by alginate and its salt forms (15.2%) occupy the second and third place at this top, respectively. The following ingredients—vitamin E and derivatives, *Aloe vera* extract, hyaluronic acid, and its salt forms, essential fatty acids, panthenol, honey, and trolamine—present usage frequencies between 5% to 15%.

**Figure 7:**
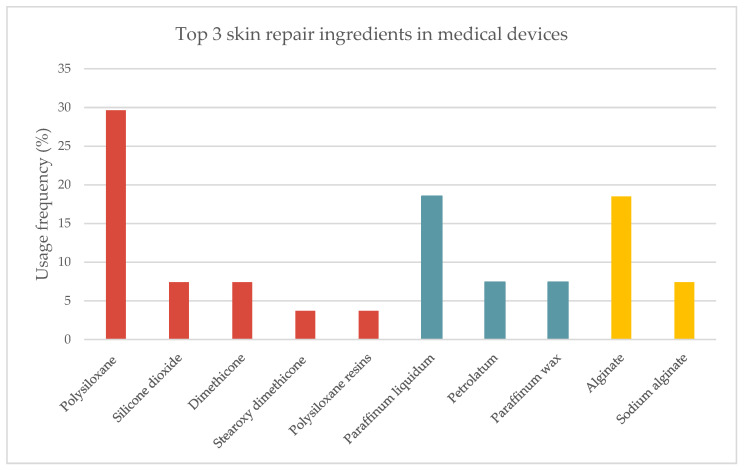
Usage frequency of the top 3 most used skin repair ingredients and actives in medical devices extended: silicones and derivatives (red), petrolatum derivatives (blue), alginate and derivative (yellow).

The top three most used ingredients underwent a detailed analysis (Figure 7). Silicones and derivatives were present in a large percentage of products. The identified silicones were polysiloxane (29.3%) along with its derivatives—silicone dioxide (7.4%), dimethicone (7.4%), stearoxy dimethicone (3.7%), and polysiloxane resins (3.7%). Petrolatum derivatives are the second most used wound regenerating ingredients, such as paraffinum liquidum (18.5%), petrolatum, and paraffinum wax (7.4% each). The third most used ingredient was alginate (18.5%) and its salt, sodium alginate (7.4%).

### 2.2. Summary of Results

More than 60 active ingredients for skin repair were found in the composition of cosmetic products whereas only nine were identified in medicines. Although the diversity of ingredients differs significantly, results show that these two categories share several common ingredients when comparing the top 10. As shown in Table 1, vitamin B5 was the only ingredient common in the three categories. *C. asiatica* and pure compounds, and metal salts and oxides were found in cosmetics and medicines, while trolamine was found in medicines and medical devices. Both vitamin E, hyaluronic acid, and their respective derivatives were found in cosmetics and medical devices. Allantoin, bisabolol, caprylic/capric triglycerides, squalene and squalene, and sunflower oil were only found in cosmetics. In parallel, birch bark extract, proteolytic enzymes, *T. vulgare*, salicylic, and vitamin A and derivatives were only found in medicines. Alginate and derivatives, aloe vera extracts, honey, petrolatum derivatives, and silicones were only found in medical devices as actives. Although they have different purposes, since topical medicines are intended to treat, and cosmetics clearly have a more aesthetic performance, both are intended to promote skin barrier integrity and reduce skin susceptibility when exposed to external agents, which might explain the use of similar actives. The different results achieved by each class can be explained either by the mechanism of action of the included actives for the concentrations of their use. Although this last information is not available it is expected to find a higher concentration of actives in medicines than in cosmetics. In fact, for cosmetics, Reg. 1223/2009 presents limits of usage for some ingredients as is the case of zinc gluconate which has a limit of 1 % (as zinc), and salicylic acid whose maximum concentration allowed is 2% in final formulation [13].

The main differences between the three health product categories lie mainly in the mechanism of action of medical devices. Although medical devices are used with the intention to treat, like medicines, they act by a distinct mechanism, mainly mechanical, which may explain the different ingredients present in the top nine. 

### 2.3. Scientific Evidence Supporting the Effectiveness of the Top Three Most Used Skin Repair Ingredients Used in Cosmetics, Medicines, and Medical Devices

**Inorganic compounds**

#### 2.3.1. Metal Salts and Oxides

**Figure 8:**
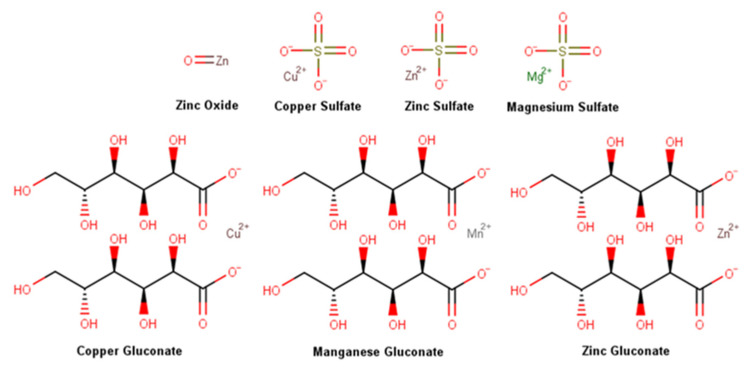
Chemical structure of the metal salts and oxides.

Metals are necessary elements for the human body, being fundamental catalytic and structural elements of proteins, enzymes, and transcription factors [26,27,28]. During the wound healing process, metals are active in the regulation of cytokines and immune mechanisms [19], and even for the intrinsic antibacterial properties of some metal elements [29]. In the pool of products analyzed, seven metal salts and oxides were highly prevalent, namely zinc oxide, the only metal oxide, and six metal salts, namely zinc, magnesium, and copper sulfate, together with copper, zinc, and manganese gluconate, the metal salt of gluconic acid, a naturally occurring carboxylic acid which increases the bioavailability of metals, whose chemical structures are featured in Figure 8. Metallostasis is highly regulated and metal ions reveal an ability to modify cell metabolism, as well as their phenotype. The role of metal elements in the regulation of wound healing and prevention of wound infection will be discussed subsequently [19].

Zinc is a metallic transitional element ubiquitously found in the body, being the second most abundant intracellular metal after iron [30]. Approximately 5% of all the zinc in the human body is stored in the skin in the form of protein complexes, where zinc acts as a cell membrane stabilizer and essential cofactor [30,31]. Zinc has been identified in more than 300 different enzymes, namely matrix metalloproteinases, a class of zinc-dependent proteins responsible for collagen fragment breakdown, which activity is enhanced in the presence of zinc oxide [31,32]. A deficiency in zinc has been associated with impaired wound healing [30,31]. In wound healing, zinc levels are increased in the inflammatory phase, revealing an even higher increase during the proliferation phase, as a result of a higher expression of membrane transporters in keratinocytes at the wound margin, macrophages, and fibroblasts. A decrease in zinc content occurs at later stages of wound healing due to cell division reduction and scar maturation [33]. In vitro, zinc sulfate did not demonstrate any effect on keratinocyte migration [34]. In human fibroblasts and keratinocytes, zinc sulfate, and zinc gluconate showed pro-proliferative and antimicrobial activities, along with anti-apoptotic features in a stimulated nutrient-deficient microenvironment [35]. Topical zinc reduced wound debris and improved epithelialization in rat surgical wounds [36]. Additionally, an in vitro assay using cultured necrotic tissue from porcine wounds confirmed that zinc oxide increases the breakdown of collagen fragments through metalloproteinases [32], boosting the expression of metalloproteinase-1 and metallothionein [37]. Zinc also enhances platelet activity and aggregation through protein kinase C (PKC)-mediated tyrosine phosphorylation of platelet proteins [38]. According to several in vivo studies, zinc oxide revealed anti-inflammatory properties [39,40,41], accelerating the wound healing process [36,39,40,41]. A study conducted in rabbits showed that topical zinc oxide accelerates wound contraction [42], while in burn treatment it increases reepithelization rate and dermis maturation, decreasing wound colonization, along with scar thickness [43]. The topical treatment with zinc gluconate healed similar results and presented a similar bacteria load when compared with other topical treatments [44]. In humans, a reduction of inflammation and bacterial growth in epidermal wounds was observed with zinc sulfate [45]. The treatment with zinc oxide accelerated non-sutured acute wound closure and significantly decreased the content of *Staphylococcus aureus* [46]. A repairing cream containing 4% zinc oxide, 2.5% dry colloidal oat extract, 0.5% oat oil, 0.2% copper sulfate, and 0.1% zinc sulfate used to treat various irritant dermatitis showed an overall rate of effectiveness superior to 82% [47]. The topical application of zinc has been shown to stimulate autolytic debridement, reduce inflammation and decrease the risk of infection, due to its antimicrobial effect. Zinc also promotes epithelialization, as a result of its involvement in cell proliferation and migration and collagen synthesis [31,48].

Magnesium is an important element in the overall human body, especially for bones and muscle strength, playing a role in over 300 enzymatic reactions and being necessary for cellular mechanisms and physiological process [49]. Magnesium possesses only one ionic form being able to coordinate with other molecules, namely sulfuric acid forming magnesium sulfate [49]. Magnesium sulfate, often referred to as Epsom salt, is the magnesium salt of sulfuric acid and can be obtained by mining as kieserite or epsomite, or by dissolving magnesium oxide, magnesium hydroxide, or magnesium carbonate in sulfuric acid [50]. Magnesium ions are small, which favors their skin penetration. They are able to decrease inflammation, enhance hydration, affect keratinocytes migration, control epidermal differentiation, increase collagen synthesis and angiogenesis, and accelerate skin barrier repair [51,52]. Moreover, magnesium blocks *N*-methyl-D-aspartate receptors, reduces intracellular calcium ions, and, consequently, has an antinociceptive effect [49]. However, only two studies were found in the literature evaluating the efficacy of magnesium sulfate in wound repair and a lack of information about its mechanism of action was noticed. The first case evaluated magnesium sulfate in patients with infected war wounds [53] and the second in ulcers [54]. In both cases, positive results seem to be achieved mainly by preventing infection. A future assay using a rat model is planned, aiming to evaluate topical agents containing magnesium sulfate in wound healing [51].

Copper is an essential nutrient for humans and around 15% of total copper in the human body is found in the skin [55]. In its pure form is a reddish-brown metallic element with the ability to assume various oxidation states, namely +2, the most abundant and stable oxidant state. Copper is obtained from ores and later is usually subject to roasting, converting, and electrolytic refining [56]. Metal copper homeostasis is required in the healing process since low levels negatively affect the process and high levels cause dysregulation and possibly trigger redox reactions, generating reactive oxygen species. The involvement of copper in the wound healing process was extensively reviewed [57]. Copper is involved in all stages of the wound healing process, performing a complex role in several cells, and modulating several cytokines and growth factor mechanisms of action. Additionally, this element is involved in the activity of platelet-derived growth factor implicated in the hemostasis phase [58,59], vascular endothelial growth factor, and angiogenin in the skin. Copper also stimulates the angiogenesis process in the proliferation phase [60,61] and the fibroblasts’ production of collagen and heat shock protein (HSP)-47, essential for collagen fibril formation. It also boosts the secretion of elastin fibber components involved in the extracellular matrix in both proliferation and remodeling phases [62,63] and assists their stabilization and the establishment of cross-linking between elastin and collagen by lysyl oxidase [64]. Modulation of integrins by keratinocytes in the remodeling phase [65] and the level and activity of matrix metalloproteinase are also modulated by copper ions [57,66], stimulating fibroblasts proliferation [66]. Copper also demonstrated biocidal activity and several mechanisms have been proposed including protein alterations and inhibition of their activity and biological assembly, membrane permeabilization, and membrane lipid peroxidation [67]. The antimicrobial effect of copper sulfate was evaluated in combination with chitosan fibers against *Staphylococcus aureus*, *S. epidermidis*, and *Micrococcus luteus*, and high effectiveness was observed [68]. A lack of in vivo studies evaluating the wound healing activity of copper sulfate and gluconate was noted. However, it is hypothesized that copper wound healing mechanisms could involve the synthesis of collagen, biocidal, anti-inflammatory, and angiogenesis effects together with epithelization promotion [69,70].

#### 2.3.2. Silicones

**Figure 9:**
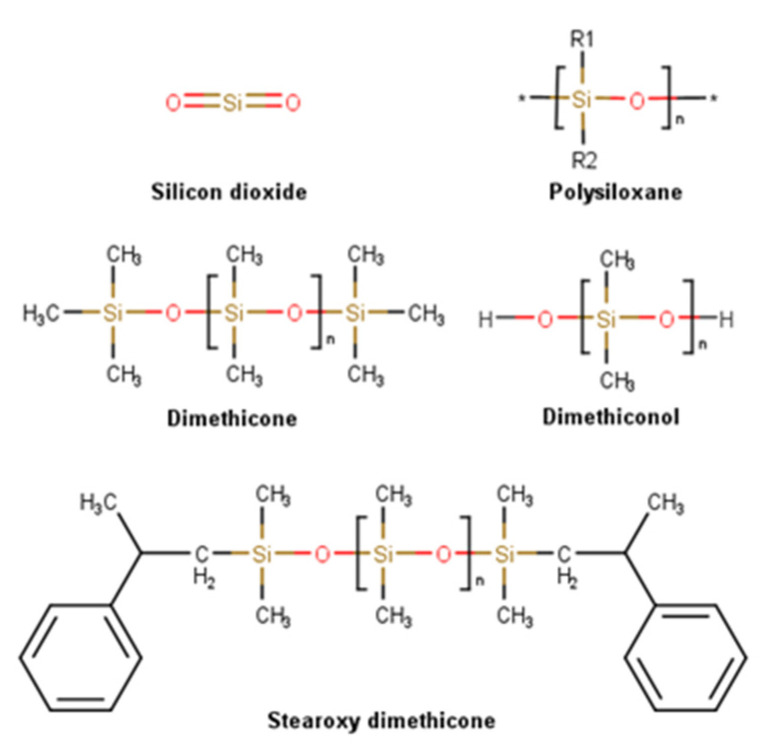
Chemical structure of silicon dioxide, polysiloxane, dimethicone, dimethiconol, and stearoxy dimethicone.

Silicones, or polysiloxanes, used in dermatology for their smoothing and occlusive properties, are polymers found in the Earth’s crust, in sand, quartz, and granite, and also in the human body [71] constituted by silicon atoms linked via oxygen atoms, forming the characteristic “siloxane” bond (Si-O-Si), with organic groups, normally methyl groups, attached to the silicon atoms. Siloxanes are building blocks forming polysiloxanes. According to their substitutions, different compounds can be synthesized, such as dimethicone or polydimethylsiloxane, with methyl groups attached to silicon atoms, and stearoxy dimethicone, similar to dimethicone but with stearoxy terminal groups (Figure 9). Silicones can be synthetized into a wide variety of materials, ranging from liquids to hard plastics, by altering the Si-O-Si chain length, side groups, and crosslinking extent [72,73]. These inorganic polymers are unique materials being used in personal care applications for more than half a century due to their distinctive properties. The resultant of the strength of the Si-O bound is a chemical with high thermal and biological stability. Their low surface tension conjugated with low viscosity and hydrophilic/hydrophobic dual behavior provide high spreadability and excellent film-forming properties improving skin hydration. When in contact with skin, which is a polar surface, the polar siloxane backbone stays in contact with the skin surface, whereas the organic groups shift outwards, resulting in a shield effect and an overall hydrophobic surface. The appreciated after-feel, a dry, light, velvety, and non-tacky sensation, is justified by the material volatility combined with high vapor pressure and low heat of vaporization, allowing quick evaporation after skin application [74]. Since silicones are non-reactive, stable, and biologically inert, they are associated with a strong safety profile [73]. Siloxane elastomers demonstrated a positive therapeutic effect when applied to the skin, in keloids, and hypertrophic scars, presumably due to their adhesive properties, ability to occlusion, and diffusion gases, such as oxygen and water vapor [75]. Silicones used in topical preparations are odorless, colorless, and nontoxic liquids [76]. Skin repair properties of silica were mainly studied in the form of nanoparticles. It was possible to verify that silica promotes platelet aggregation [77,78], enhancing the adhesion and proliferation of fibroblast cells [79], contributing to the adsorption and controlled release of keratinocyte growth factor [80].

Silicone has been widely used in the treatment and prevention of hypertrophic and keloidal scars, especially after burns and following surgical procedures [81]. After the application of silicone gel sheeting, scars become flatter, softer, and more flexible [81,82,83,84,85], regardless of how long they have been formed. Silicone gel also demonstrated promising results in the prevention of hypertrophic scar development in sternotomy wounds, without demonstrating side effects, therefore highlighting their possible use in other surgeries to minimize the formation of hypertrophic scars [81]. In a randomized comparative trial, patients received three sessions of Erbium:YAG laser, a skin resurfacing laser Er:YAG, to treat acne scars and applied a silicone gel composed by cyclopentasioloxane incorporating vitamin C ester or a hydrophilic cream to their assigned half-face. A significant reduction in roughness was observed with topical silicone, without observing significant differences in smoothness, hydration, or TEWL when compared with the placebo group [86]. In another study where a silicone gel was applied after patients underwent Erbium:YAG fractional laser resurfacing, a reduction in erythema and hyperpigmentation was observed [87]. De Giorgi et al. [88] also demonstrated silicone gel’s ability to reduce the formation of keloid and hypertrophic scars, as well as signs/symptoms associated with the wound healing process, namely color alterations, pulling sensation, and paraesthesia.

Even though the exact mechanism of action behind silicone’s wound healing properties remains unknown, several explanations have been proposed. It is believed that the SC is the main target of silicone, acting as a barrier, thus allowing skin respiration while reducing water vapor transmission, which ultimately results in increased wound moisture thus restoring homeostasis [84]. Stratum corneum was hypothesized to function as a water reservoir [89]. The cellular effects of silicone and hydration were analyzed in vitro using keratinocytes and fibroblasts, where it was found that hydration provided by silicone modulates the effects on the cells under study. In fact, this study supports the hypothesis that hydration or water impermeability may be responsible for its effective healing results and the inhibition of fibroblasts proliferation, together with inhibition of collagen and glycosaminoglycan production in the hydrated group and no apparent change in fibroblast activity in the silicone-treated group [90]. Silicone also causes immunological alterations, namely downregulating fibrogenic cytokine TGFβ2 [91], TGF-β [92], or increasing basic fibroblast growth factor and IL-8 levels [93,94]. Since silicone is inert, it is not capable of inhibiting microbial growth, but it can act as a bacterial barrier [81]. It is also associated with a reduction of mast cell activity, edema, and excessive extracellular matrix formation. Silicone also contributes to the right alignment of collagen deposition by increasing temperature, oxygen tension, and electrostatic properties [81,92].

**Hydrocarbon compounds**

#### 2.3.3. Petrolatum Derivatives

**Figure 10:**
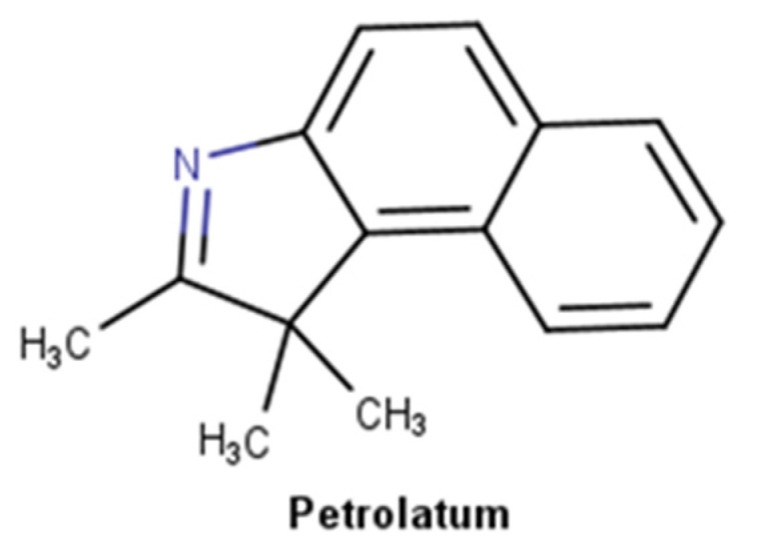
Chemical structure of petrolatum.

Petroleum derivatives are complex substances (Figure 10) that constitute a wide group of materials prepared from crude oil by distillation, often followed by other treatments to remove undesired constituents, namely sulfur and unsaturated aromatic compounds [95]. The two most important materials for dermatology for their emollient and occlusive properties are liquid paraffin, also known as mineral oil and paraffinum liquidum, and petrolatum, commonly known as Vaseline, that result from a complex combination of hydrocarbons [96]. All petrolatum derivatives are mixtures of hydrocarbons: petrolatum is a purified mixture of semisolid saturated hydrocarbons with branched and unbranched chains, and sometimes cyclic alkanes and aromatic molecules, with the general formula C_n_H_2n+2;_ paraffin is a mixture of solid saturated hydrocarbons with the same general formula of petrolatum; and mineral oil is a mixture of liquid saturated aliphatic, between 14 to 18 carbons, and cyclic hydrocarbons [97]. Petrolatum was incorporated in the pharmaceutical and skincare industry firstly as a vehicle and nowadays as an effective moisturizer ingredient. This mixture of hydrocarbons creates an oily barrier over the skin surface, reducing TEWL both in healthy [98] and irritated human skin [99,100]. Due to its occlusive properties, petrolatum derivatives can retain water, consequently restoring the skin barrier. Petrolatum is an inert substance, meaning that it cannot bind to proteins or undergoes cutaneous chemical alteration [101]. Additionally, it is not absorbed through intact or injured skin and is not sensitizing or irritating [102]. Petrolatum in the minimum concentration of 5% reduces TEWL by more than 98%, superior to lanolin, mineral oil, and silicones which only reduce between 20–30% [96].

The efficacy of petrolatum in the treatment of auricular wounds was evaluated proving to be an effective wound care ointment with a possible association with anti-inflammatory properties [103]. In the treatment of wounds in the neck of horses, petrolatum-treated wounds developed excessive granulation tissue, possibly due to the occlusive properties of petrolatum which creates a low oxygen tension, resulting in excessive granulation tissue growth. Moreover, petrolatum-treated wounds accumulated significantly more bacteria when compared with control, while presenting a higher healing rate [104]. When compared with a prescription medical device oil-in-water emulsion indicated for the treatment of superficial wounds, a petroleum-based treatment demonstrated superior efficacy in wound healing [105], as well as greater efficacy in preventing diaper rash [106]. In spite of accumulating bacteria colonies in wound sites, petrolatum has been widely proposed to prevent surgical skin infections [107]. Several studies comparing infection rates between wounds treated with topical antibiotics and petrolatum-based products showed no difference [103,107,108,109,110]. Therefore, Saco et al. [111] proposed petrolatum-based products to be used instead of topical antibiotics in the prevention of postsurgical infections. In another study, in the treatment of wounds created by the removal of dermatosis papulose nigra papules in an African American population, a petrolatum-based ointment demonstrated equivalent safety and efficacy and improvement of wound appearance when compared with an antibiotic-based polysporin ointment [112].

Petroleum-based wound healing products’ mechanism is based fundamentally on their occlusive properties. By creating a hydrophobic barrier, petrolatum prevents evaporative water loss creating an optimal environment for restoring stratum corneum barrier [113]. By keeping the wound hydrated, angiogenesis, collagen synthesis, dead tissue, and fibrin breakdown are increased, and scab formation is prevented [114]. Moreover, it significantly upregulated innate immune genes and antimicrobial peptides, and increased epidermal differentiation [115]. It is also demonstrated to have equal properties to prophylactic topical antibiotics in preventing infections after clean cutaneous procedures [103,107,108,109,110].

**Vitamins**

#### 2.3.4. Vitamin E and Derivatives 

**Figure 11:**
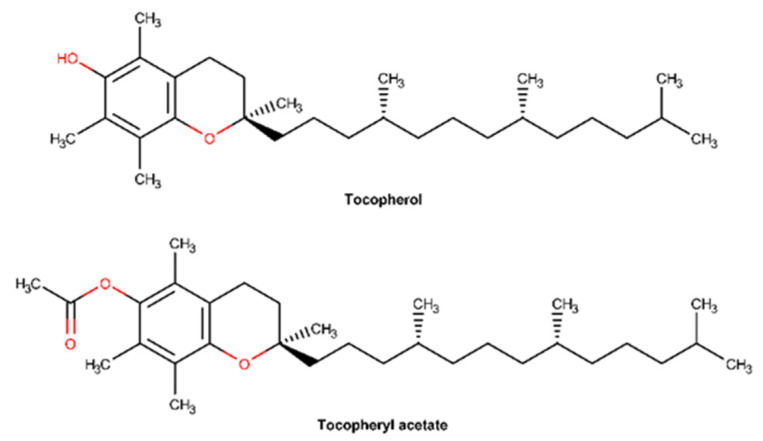
Chemical structure of tocopherol and tocopheryl acetate.

Vitamin E is the major lipophilic compound, commonly found in fruits, vegetables, and seeds, present in plasma, membranes, and tissues and with strong antioxidant activity [116,117]. Vitamin E is the major lipid-soluble antioxidant in the skin, being rapidly absorbed and integrated within the lipidic skin layer, supporting the extracellular lipid matrix of the stratum corneum and contributing to enhancing its antioxidant defenses [118]. It is widely used in cosmetics due to its ability to scavenge free radicals, namely hydroxyl radical, singlet oxygen, and superoxide anion [119,120]. The generic term vitamin E includes all forms of tocopherols and tocotrienols. Eight naturally occurring structural analogs of vitamin E are known, four tocopherols (α-, β-, γ-, and δ-analogs) and four tocotrienols (α-, β-, γ-, and δ-analogs) which differ in the number and position of methyl groups on the chromanol ring [121]. Among all, the stereoisomer α-tocopherol is the predominant form of vitamin E in nature and in the human body and has a potent biological activity [122,123]. Nonetheless, α-tocopherol is photo and chemically unstable, vulnerable to oxidation by alkoxyl radicals, which compromises its activity. Therefore, tocopheryl acetate was developed and is often preferred in topical formulations due to its higher stability. It is enzymatically converted by skin esterases into the active form (α-tocopherol) [117]. Both tocopherol and tocopheryl acetate (Figure 11) are frequently used in dermatological practice to increase wound healing rate, prevent hypertrophic scaring, and reduce pruritus, along with moisturizer skin effects [123,124].

Several studies highlighted that the protective properties of vitamin E on skin repair are primarily due to its antioxidant, anti-inflammatory, and moisturizing effects. In fact, a recent single-blinded study performed on children revealed that topical vitamin E, before and after surgery, improved surgical wound healing and improved cosmetic results [125]. The administration of α-tocopherol acetate to female Wistar rats reduced the inflammatory processes and increased the repair process of induced lesions on irradiated mucosa [126]. Topical vitamin E can reduce UV-induced skin swelling, skin thickness, erythema, and edema, all signs of skin inflammation. In mouse skin, α-tocopheryl acetate significantly inhibited UV-induced iNOS mRNA expression, NO production, and COX-2 activity, while reducing PGE2 levels, so limiting inflammatory response evoked by UV radiation exposure [127]. α-Tocopheryl acetate treatment of superficial burns, post-traumatic superficial ulcers, and skin graft donor sites also contributed to a quicker reduction of exudates and pain, along with a progressive and faster wound healing [128]. The topical treatment after sinonasal surgery in elderly patients with α-tocopherol acetate showed a higher improvement and increase in the restoration of sinonasal mucosa when compared with topical treatment with nasal hypertonic saline solution and gomenol oil [129]. The moisturizing properties of vitamin E have been the subject of some in vitro experiments. Several studies revealed that topical application of vitamin E reduced the TEWL and improved the ability of the skin to retain water, thus favoring hydration and reestablishing the skin barrier function [124,130].

Through its scavenging activity, vitamin E protects cell membranes and polyunsaturated lipids from reactive oxygen species (ROS) attack by inducing the activation of various signal transduction pathways and is thus recognized mostly for its role as an antioxidant [131]. Tocopherol has the ability to interfere with lipid peroxidation of cell membranes, acting as a “chain breaker” by scavenging peroxyl radicals [117]. Since inflammation is one of the phases of the wound healing process, characterized by a major production of ROS and reactive nitrogen species (RNS); tocopherol’s ability to neutralize these oxidative agents may accelerate the skin repair process. Vitamin E also modulates the expression of connective tissue growth factor [131], while enhancing both collagen synthesis and preventing collagen degradation, thus preserving the skin barrier integrity [20,117,132].

#### 2.3.5. Dexpanthenol

**Figure 12:**
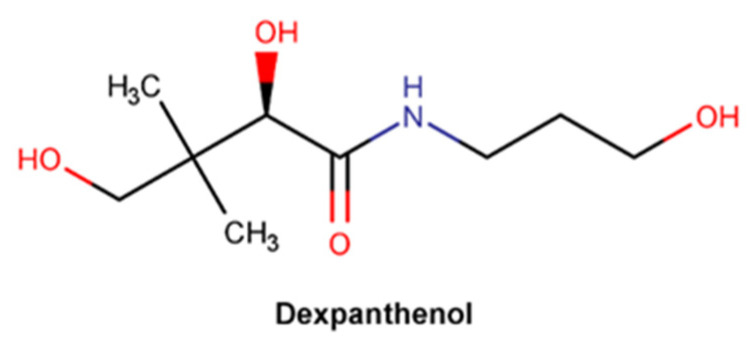
Chemical structure of dexpanthenol.

Dexpanthenol, chemically known as 2,4-dihydroxy-*N*-(3-hydroxypropyl)-3,3-dimethylbutanamide (Figure 12), is the dextrorotatory eutomer of panthenol (D-panthenol), the only biologically active form (133). D-panthenol is an analogue of pantothenic acid (vitamin B5), that is often preferred in topical formulations due to its high stability [133,134,135,136]. Pantothenic acid is a key precursor to coenzyme A biosynthesis, a co-factor essential to cell growth and both synthesis and oxidation of fatty acids, vital to the stratum corneum integrity [134,135,137]. D-panthenol readily penetrates through the skin and is enzymatically converted into pantothenic acid. Hence, dexpanthenol presents a dual action profile: as a moisturizer and humectant, due to its high hygroscopicity, and as a wound healing facilitator agent, promoting epidermal regeneration [134,135]. 

Many clinical studies provide experimental evidence of the beneficial effects of topically applied dexpanthenol. A randomized, double-blind, vehicle-controlled study was performed to evaluate the effect of topical dexpanthenol on epidermal barrier function and SC hydration [136]. The results achieved demonstrated the formulation considerably reduced TEWL, increasing the SC water content, and subsequently improving the SC hydration [136]. Another three open-label, randomized, intraindividual comparison studies between three different emollient formulations with the same concentration of dexpanthenol in healthy adult subjects with dry and sensitive skin confirmed that the formulations significantly improve skin elasticity parameters with high cutaneous tolerance [138]. Formulations containing dexpanthenol contribute to smoothing and hydrating injured skin, after tattooing procedures, therefore reinforcing the ability to maintain the skin barrier integrity [139]. Furthermore, in comparison with petrolatum jelly, topical dexpanthenol promotes skin hydration without the formation of acneiform lesions, often developed due to the occlusiveness provided by formulations with petrolatum derivatives [140]. It was also confirmed that these long-lasting skin-moisturizing effects (over 24 hours), as well as a relocation of the water molecules into deeper layers of the SC [141], do not negatively impact the cutaneous microflora viability [142]. However, an experimental study with excised porcine skin showed that dexpanthenol also promotes skin hydration through the increase in molecular mobility of several lipid and protein segments of the SC [143]. The improvement of skin inflammation typical signs, namely redness and roughness, was confirmed by two different clinical trials whose aim was to understand the effect of a dexpanthenol-containing cream on skin barrier after sodium lauryl sulfate (SLS)-induced irritation [144,145]. A randomized, double-blind, single-center, placebo-controlled study revealed that dexpanthenol highly impacts the proliferation of dermal fibroblasts through upregulation of *IL-6*, *CYP1B1*, *CCL2*, and *CXCL1* gene expression, modulating gene expression related to the wound healing process [146]. Several in vitro studies also corroborate the impact of dexpanthenol in human fibroblasts, enhancing cellular migration, attachment of fibroblasts, and collagen synthesis [135].

The mechanisms of action by which dexpanthenol accelerates skin barrier repair, increases skin hydration, reduces skin’s inflammation and roughness, and promotes wound regeneration are only partially elucidated [144]. Dexpanthenol may improve skin hydration directly due to its high hygroscopicity, promoting moisture content retention. Additionally, D-panthenol was also found to increase the interaction with lipid segments of the extracellular lamellae and protein residues in the SC corneocytes, therefore boosting molecular mobility/fluidity [138,143]. By this mechanism, dexpanthenol can directly compensate for the reduced hydration and increased rigidity of the SC lipid lamellae and keratin filaments, characteristics of dry skin [138,143]. On the other hand, D-panthenol may promote coenzyme A production and, consequently, acetyl CoA, an essential player in the fatty acid and sphingolipid synthesis reaction cascade. Thus, it contributes indirectly to skin hydration, replacing the essential lipids composition and, therefore, restoring SC integrity [144]. Dexpanthenol’s ability to increase fibroblast proliferation and accelerate epithelialization [135] may beneficially impact the regeneration of both deep and superficial wounds.

#### 2.3.6. Vitamin A and Derivatives

**Figure 13:**
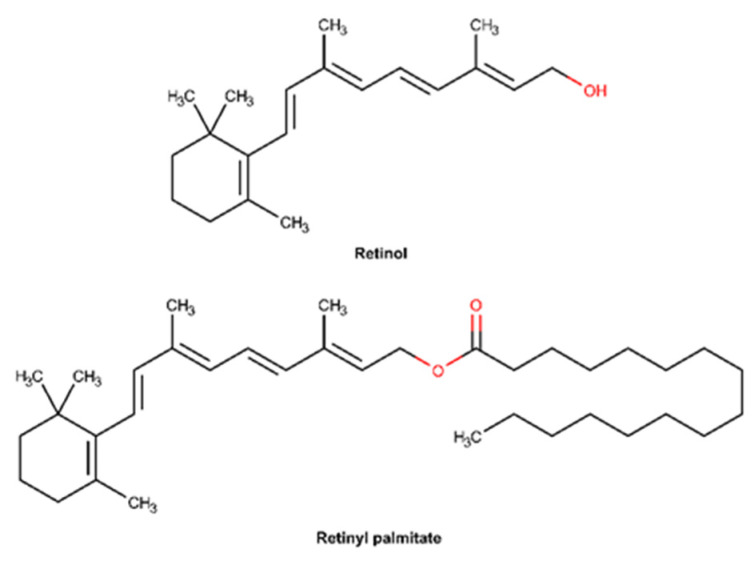
Chemical structure of retinol and retinyl palmitate.

Vitamin A constitutes a group of lipid-soluble compounds including retinol and retinyl palmitate, whose chemical structures are shown in Figure 13 [147]. Retinol is the main circulating form of the vitamin in the body, whereas retinoic acid is the main active metabolite [148]. Retinol is composed of a non-aromatic β-ionone attached to an unsaturated isoprenoid side chain with a hydroxyl group in the final chain [149]. Retinyl palmitate is an ester of retinol and is the major form of vitamin A found in the epidermis [150]. This compound is often used in formulations instead of retinol due to its higher stability [150]. The bioactivation of topically applied retinyl palmitate depends on its conversion to retinoic acid, which requires an enzymatic cleavage of the ester bond by skin esterases and subsequent NAD+ dependent oxidation [147,150,151]. Retinoids are known to be critical in maintaining epidermal homeostasis, particularly regulating proliferation and differentiation [147]. In wounded tissue, vitamin A stimulates epidermal turnover, increases the rate of re-epithelialization, and restores epithelial structure [152].

There are several studies confirming that topical application of retinol significantly affects cellular and molecular properties of both the epidermis and dermis [153]. In fact, a randomized, double-blind, vehicle-controlled, left and right arm comparison study performed on 36 elderly individuals revealed that retinol improves the clinical appearance of fine wrinkles, restoring the skin barrier by increasing glycosaminoglycans and collagen synthesis [154]. Several other in vivo studies also corroborate the tensile properties of vitamin A, namely through the reduction of matrix-degrading proteins and increasing cell growth [155,156,157]. Vitamin A was found to induce epidermal thickening, with a high tolerability profile [153,158,159]. Likewise, there is scientific evidence demonstrating that vitamin A has an active role in the wound healing process. An in vitro study performed in mouse skin normal fibroblast, human umbilical vein endothelial cell, and monocyte/macrophage-like cell line revealed that vitamin A decreases the nitric oxide production, an inflammatory mediator, in a dose-dependent way, exhibiting anti-inflammatory activity [160]. Therefore, vitamin A accelerates the resolution of the inflammatory phase of wound healing, contributing to a rapid skin barrier repair. Another in vitro study suggests that vitamin A enhances collagen synthesis and fibroblast differentiation, stimulating the wound healing process [161]. 

In summary, it is suggested that retinol interacts with receptors inside keratinocytes, promoting cellular proliferation, strengthening the epidermal protective function, reducing transepidermal water loss, protecting collagen’s degradation, and inhibiting the action of metalloproteinases, whose main goal is to degrade the extracellular matrix [147,156,162,163]. Moreover, vitamin A promotes the remodeling of reticular fibers and stimulates angiogenesis in the papillary layer of the dermis, promoting endothelial cell migration [149,160]. Vitamin A’s ability to mitigate the inflammation phase, by decreasing nitric oxide production, is believed to be the mechanism that explains the acceleration of the wound healing process [160].

**Botanic extracts**

#### 2.3.7. *C. asiatica* and Pure Compounds

*C. asiatica* is a medicinal plant also known as Gotu Kola, with reported wound healing properties. Due to its traditional use, mainly in Asia, its monography was included in the European Pharmacopoeia during the 19th century [164].

**Figure 14:**
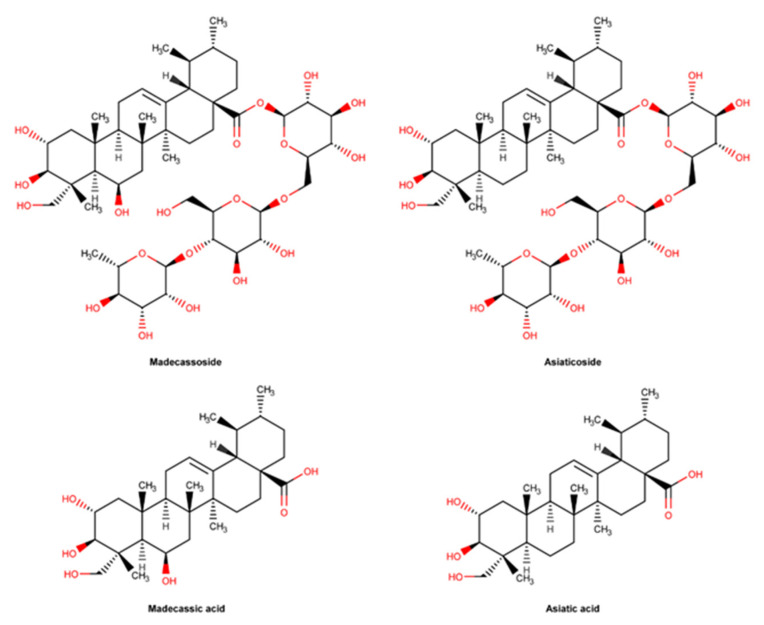
Chemical structure of madecassoside, asiaticoside, madecassic acid, and asiatic acid.

The major components of *C. asiatica* with pharmacological activity are asiaticoside, madecassoside, asiatic acid, and madecassic acid as displayed in Figure 14. These pentacyclic triterpenes, also known as centelloides, are bioactives with demonstrated wound healing activity [165]. They can be extracted from *C. asiatica* in different percentages depending on the part of the plant and solvent of extraction, time of harvesting or geographical area [165,166]. Asiaticoside and madecassoside are pentacyclic triterpenes with a glycosidic structure, often used as extract biomarkers [167,168]. Their structure includes in the ursane scaffold three hydroxyl groups, an olefin and a carboxylic acid [169]. As shown in Figure 14, asiatic and madecassic acids are the corresponding aglycones forms of asiaticoside and madescassoside, respectively. In acidic conditions, asiatic acid can be successfully formed through the hydrolysis of the sugar portion of asiaticoside. *C.asiatica* can be incorporated in the formulation as the whole extract or as the selected bioactive [166].

*C. asiatica* is widely used due to its antioxidant, anti-inflammatory, and wound healing properties [166]. A randomized, controlled, double-blind trial performed in 30 patients to evaluate the efficacy of a cream containing *C. asiatica* confirmed its ability to improve scar appearance [170]. An in vivo study performed with male Sprague Dawley rats confirmed the effect of different *C. asiatica* extracts in wound healing, and the asiatic acid bioactive was the most effective [171]. Asiatic acid shows promising activity in the prevention of keloids by inhibiting TGF-1β -induced collagen and plasminogen activator inhibitor (PAI-1) expression, which ultimately results in the effacement of collagen deposition in fibroblasts [169]. Another in vivo study using a gel with *C. asiatica* standardized extract, containing madecassoside at 51% and asiaticoside at 38%, when applied after laser treatment, demonstrated facial acne scar improvement [172]. Asiaticoside also increased the activation of antioxidant levels at an early stage of the healing process through the regulation of the Smad pathway, via the transforming growth factor (TGF) β1 receptor, attenuating ROS generation and superoxide dismutase (SOD) reduction [173,174]. Through the modulation of the same via, asiaticoside reveals the ability to inhibit the growth of keloid hypertrophic scars [175]. Several in vitro studies have confirmed that asiaticoside stimulates wound repair by increasing collagen, fibroblast proliferation, and extracellular matrix (ECM) synthesis [176,177]. Furthermore, this compound promotes angiogenesis by enhancing the levels of relevant cytokines such as monocyte chemoattractant protein-1 (MCP-1), VEGF and IL-1β [178]. Similarly to asiaticoside, madecassoside significantly favors wound healing by several mechanisms, namely anti-inflammatory and antioxidant effect, collagen synthesis, and by promoting angiogenesis, as demonstrated in burn injuries [179]. 

The traditional use of *C. asiatica* over the years is widely supported by scientific evidence, although studies are scarce when concerning the sole use of either madecassoside or madecassic acid.

**Marine ingredients**

#### 2.3.8. Alginate and Derivatives

**Figure 15:**
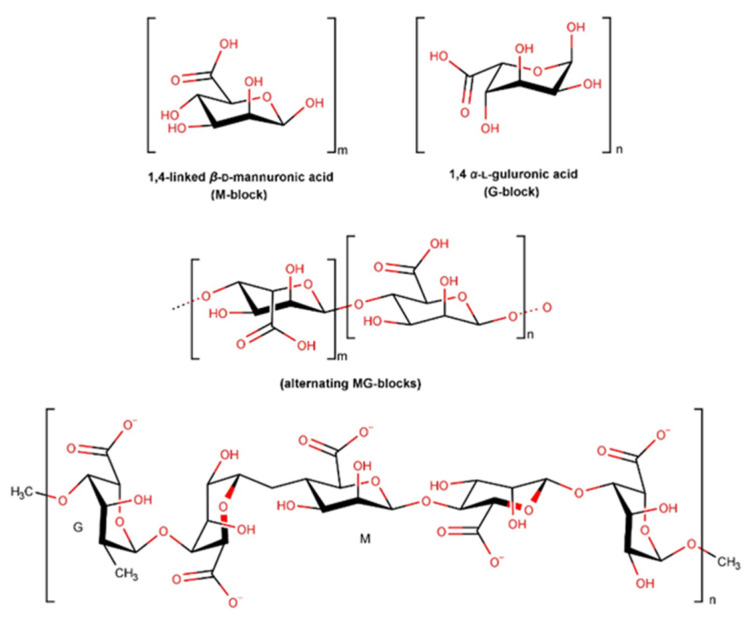
Chemical structure of alginate and respective monomers.

Alginate is a naturally occurring polysaccharide, mainly obtained from the cell wall of brown seaweeds [180]. As shown in Figure 15, alginates are formed by β-D-mannuronic (M) and α-L-guluronic (G) acid units that form regions of homogeneous M-blocks and G-blocks as well as heterogeneous blocks of alternating sequences (MG blocks). The configuration of this structure is dependent on the alginate’s source, part of the plant used, or harvesting season, leading to a wide structural variation, different molecular weights, and physicochemical properties. The conversion of alginates into esters and monovalent salts such as calcium and sodium promotes their water solubility. Sodium alginate represents alginate’s most common salt [181]. Its high swelling capacity promotes a weak jellification in the wound environment, promoting moisturization and epidermal regeneration. This property is of special importance in the management of exudative wounds [182]. The biocompatibility and biodegradability of alginates make them very appealing ingredients for pharmaceutical applications [181,183]. Alginate is mostly used in the form of a hydrogel. Hydrogels are water-soluble cross-linked polymeric networks formed by physical or chemical cross-linking of soluble polymers [184]. 

Several studies have shown that alginate plays an important role in preventing infection in wound environments through its anti-infectious properties as well as antioxidant and anti-inflammatory effects promoting skin regeneration [182]. An in vivo study revealed that the use of alginate accelerates wound healing when compared to petrolatum. Decreased levels of TGF- β1, fibronectin, and VEGF were demonstrated, while levels of collagen-I increased [185]. An in vitro study confirmed that either mannuronic or guluronic groups present in alginates have a potential prothrombotic effect, however via different pathways. Whilst M-block alginates were able to extend thrombin formation and the intrinsic pathway, G- blocks and mixed MG-blocks appeared to affect the fibrin polymerization [186]. Despite the several studies performed with alginates in wound healing, there is a lack of information on their mechanism of action. Thus, their high absorption rate, limiting wound secretion, and minimization of bacterial contaminations appear to be the main reason for the alginates’ use [185]. 

## 3. Materials and Methods

### 3.1. Data Collection

Data were collected from wound repair health products marketed in pharmacies and parapharmacies (commercial facilities similar to a pharmacy, also requiring a pharmacist as technical director, but that cannot sell prescription medicines) in Portugal during 2022, namely cosmetics, topical medical devices, and topical medicines. 

Cosmetic product information was collected from “Farmácias Portuguesas” website and major online pharmacies’ websites. Cosmetics were included in the study if they exhibited one of the following terms in the label: cica, scar, wound, repair/repairing, or regenerating. Cosmetics for application on the face, lips, body, hands, and feet for both adults and babies were considered. Face and body cleaning products were also analyzed, while sunscreens and after-suns were excluded. Following the inclusion criteria 120 cosmetic products, from 43 international cosmetic brands were included. Labels and compositions were collected from the product’s label and/or manufacturer’s website.

Medical devices were selected based on a Code List from INFARMED (Portuguese health products regulatory authority) website, downloaded on 2 November 2022 (https://www.infarmed.pt/web/infarmed/pesquisa-dispositivos). Products were browsed from the Code List if they were registered on the following Portuguese Nomenclature of the Medical Device codes: M0499 (Special dressings—others), M04040702 (Silicone dressings associated with other substances), M040499 (Dressings for wounds, injuries, and ulcerations—others), M9099 (Medication Devices—others), M040413 (Bioactive dressings), V9099 (Devices not included in previous classes—other) and M040199 (Prepared dressings—others). Only topical formulations (cream, gel, lotion, liquids, ointments, and sprays) were selected and included if one of the following terms was found on the label: cica, scar, wound, repairing, regenerating. Gauze, sterilized absorbent cotton, and bandage dressings were excluded due to a lack of information about their composition. Label information of 46 medical devices was collected from INFARMED’s website. 

Medicines were selected from the Portuguese database of medicines for human use, INFOMED on 16 November 2022 (https://extranet.infarmed.pt/INFOMED-fo/index.xhtml) from the category “13-Medicines used in skin conditions” of Pharmacotherapeutic Classification, based on their function resulting in the inclusion of 21 products.

### 3.2. Data Analysis

#### 3.2.1. Skin Repair Ingredients or Active Substances Prevalence

The relative number of cosmetics, topical medicines, and medical devices containing skin repair ingredients or active substances was determined and expressed in percentage.

#### 3.2.2. Top of Most Used Skin Repair Ingredients or Active Substances

A preliminary analysis was performed to identify the top 10 most used wound repair ingredients or actives in cosmetic products, topical medicines, and medical devices. Subsequently, an in-depth analysis focused on the top 3 wound repair ingredients or active substances with the highest usage frequency in cosmetic products, medicines, and medical devices was performed. The usage frequency for each ingredient or active substance was expressed in percentage and the ingredients were ranked in descending order. To analyze the occurrence of each ingredient in the pool of products collected from each category, absolute and relative frequencies were calculated.

#### 3.2.3. Scientific Evidence for Skin Repair Ingredients or Active Substances

The scientific evidence supporting the skin wound repair effect was searched for the top 3 most used wound repair ingredients found in cosmetic products, medicines, and medical devices, on the following online databases: PubMed, Google Scholar, Scopus, Cochrane, and KOSMET. The search was performed, using the keywords ((“INCI name” (Title/Abstract) OR “synonyms”, when applicable) AND (skin(Title/Abstract) OR topical(Title/Abstract)) AND (wound(Title/Abstract)) OR healing OR repair* OR regenerate*(Title/Abstract)).

## 4. Summary of the Mechanisms of Action

All the mechanisms of action of the ingredients that are present in the top 3 most used ingredients in cosmetics, medicines, and medical devices with proven skin repair effects are summarized in Table 2.

Metal salts and oxides contribute to the acceleration of the wound healing process by interfering in the inflammatory phase, along with their anti-microbial effect, both in cosmetics and medicines. Since zinc, copper, and manganese are components of the antioxidant enzyme superoxide dismutase (SOD), they can decrease inflammation through the neutralization of ROS and other peroxides generated in the body, protecting the integrity of cell membranes [1]. In addition, zinc and copper promote epithelialization and collagen synthesis, while magnesium ions also affect keratinocytes migration epidermal differentiation, and reduce intracellular calcium ions, revealing an antinociceptive effect. On the other hand, vitamin E accelerates the wound healing process essentially because of its antioxidant effect, neutralizing ROS and RNS, whose production is increased during the inflammatory phase. Additionally, tocopherol enhances collagen synthesis, while preventing collagen degradation, through the modulation of the expression of connective tissue growth factor. *C. asiatica* is a major botanical ingredient present in both cosmetics and medicines top 10. This extract, as well as its major active ingredients, asaticoside, madecassocide, asiatic and madecassic acid, displays effective wound healing properties by inhibiting inflammation, inducing collagen synthesis, promoting angiogenesis, and reducing wound oxidative stress. Both vitamin A and dexpanthenol promote skin repair by promoting skin hydration, accelerating the re-epithelialization process. Vitamin A interacts with the retinoic receptors located inside the keratinocytes, promoting cell proliferation, and decreasing TEWL. On the other hand, dexpanthenol’s hygroscopicity allows the retention of water molecules, reducing TEWL, while boosting the synthesis of coenzyme A, which is necessary for the synthesis of lipids structurally required for the SC and, as a result, helps to rebuild the skin barrier. Furthermore, vitamin A also interferes with the inflammatory phase of wound regeneration by decreasing the production of nitric oxide, a pro-inflammatory mediator. As far as silicones and oil derivatives are concerned, the mechanism of action promoting wound healing consists of a barrier effect caused by the deposition of a film which consequently leads to a decrease in TEWL. In addition to the moisturizing effect, petroleum derivatives also show antimicrobial properties. Alginate has a dual mechanism of action since it forms a film that prevents TEWL and simultaneously has the ability to absorb exudates from the wound site, creating a moist environment that promotes the healing process. 

## 5. Conclusions

Skin injuries result from the disruption of the epidermal and/or dermal barrier, thus predisposing them to an increased TEWL, while enabling the entry of microorganisms. Some underlying pathologies that are predisposed to the fragility of the cutaneous barrier may compromise self-repairing mechanisms. Therefore, in order to improve the skin repairing processes, a plethora of cosmetics, medicines, and medical devices are available in the market.

In order to characterize the different available skin repair strategies, an up-to-date overview of the most used skin repair ingredients was performed enrolling 120 cosmetic products, 21 medicines, and 46 medical devices, available in the Portuguese market. Furthermore, scientific evidence regarding the effectiveness of skin regenerating properties and the respective mechanisms of action was also collected. Three different tops were conceptualized since cosmetic products, medicines, and medical devices are subject to different regulations and consequently have different purposes.

While cosmetic products feature an average of four or more ingredients with skin-repairing properties in each formulation, in drugs and medical devices mainly one was identified. In fact, more than 60 active ingredients for skin repair were found in the composition of cosmetic products which is clear evidence of the variety and innovation of this industry. The glossary of common ingredients complies with more than 30.000 entries of common ingredient names that are authorized to be included in cosmetic formulations. Medicines and medical devices’ claims concerning curative properties can only be made for the active substance(s), whose demonstration of efficacy must be previously confirmed by the regulatory authorities. Moreover, these products are subject to more conservative safety assessments than cosmetic products, which restricts the range of ingredients that can be used. Indeed, the raw materials used for medicines development must be listed in Pharmacopoeias or other official documents, a criterion that sets a clear restriction on the number of ingredients that can be used. Conversely, cosmetics comprise a great and diverse global market, whose growth is highly dependent on new ingredients and technological innovations. 

The differences between the three health product categories depend mainly on the mechanism of action of medical devices. Although medical devices are used with a therapeutic purpose, like medicines, they act by a distinct mechanism, mainly physical which may explain the different ingredients present in this category. Wound repairing action by medical devices results essentially from ingredients that provide a decrease of TEWL by occlusion. On the other hand, the action of topical medications/cosmetics in decreasing TEWL is achieved by increasing the production of lipids essential to the SC structure and the use of emollients. 

Some of the ingredients used in cosmetics coincide to with those used in medicines, and thus the skin-repairing action is justified by similar mechanisms of action. However, it is known that, although the ingredients may sometimes be the same (like zinc oxide, present in both cosmetics and medicines top 3) the concentration in which they are found in cosmetics and medicines may differ. Regarding the site of action, usually, the main target skin layer for cosmetics is the epidermis while for medicines an action on both the epidermis and dermis are to be expected. That is the case of dexpanthenol and vitamin A and respective derivatives, present in the top 3 of medicines, but not on cosmetic products, which can directly repair and restore both the barrier function and dermal matrix. On the other hand, vitamin E and derivatives and *C. asiatica* and respective pure compounds improve moisturization, decrease inflammation and restore dermal matrix and are widely used in cosmetics. Thus, in this category, cosmetic products exhibit their skin repair activity by acting on both skin layers.

The increasing knowledge of the advantages of substances derived from plants is one of the main factors driving the market for natural skin care products. In fact, skin repair can be facilitated by natural products which have revealed anti-inflammatory, antioxidant, antibacterial, and pro-collagen synthesis properties. Both in medicines and cosmetics a natural extract (*C. asiatica*) was used but to a greater extent in the latter, thus confirming the trend of natural cosmetics. 

The antimicrobial effect seems also to be a key feature of skin repair formulations, either afforded by metal salts and oxides (both in cosmetics and topical medicines) or by improving the barrier and preventing bacteria entry (medical devices). Therefore, both cosmetics and topical medicines can be used to protect and strengthen the skin barrier, repair the skin, and restore its function, either at a superficial or dermal level, but should be selected depending on the type and depth of the lesion. Noteworthily, cosmetics should only be used on skin without open injuries and in mild conditions. 

In conclusion, this study identified current strategies to promote skin repair and the respective mechanisms of action. Scientific evidence on the top three most used ingredients of medical devices is much scarcer than the top three ingredients of medicines and cosmetics, so more in vivo studies should be conducted in order to enlighten their effectiveness in the wound healing process. This work highlights the differences between the composition of cosmetics, medicines, and medical devices, providing a useful tool for helping health professionals to make an informed decision concerning the selection of a topical skin repair formulation. This insight is also useful to identify opportunities for innovation in the chemical and cosmetic industry.

## 6. Strengths and Limitations

This work only considered cosmetic products available in Portuguese pharmacies and parapharmacies, as well as medicines and medical devices, currently commercialized in Portugal, authorized by INFARMED, the regulatory authority in Portugal responsible for the supervision of all the mentioned health products. This work resulted from a critical, comprehensive, and exhaustive analysis of the composition of all cosmetics, medicines, and medical devices collected until November 2022, providing a wide overview of the available commercial products for this subject. However, the impossibility to access the concentrations of active ingredients in cosmetics and medical devices may represent a limitation of the study. Moreover, information is sparse about the category classification of the formulations used in studies, since usually they are only classified in later stages of product development. 

Market comparison with other countries was not found. However, the Portuguese pharmacy and parapharmacy cosmetic market is dominated by multinational cosmetic brands from international corporations. Therefore, these results could cover a significant portion of the market in other European countries. The absence of in-house studies, particularly those promoted by cosmetics industries, was another problem that limited the search for scientific evidence. Even though this work focused on the analysis of the ingredients in cosmetics, topical medicines, and medical devices that were avowedly actives, the entire composition may contribute to its desirable properties.

## Figures and Tables

**Table 1:** Summary of the ingredients found in cosmetics, medicines, and medical devices.

Ingredient	Cosmetics	Medicines	Medical Devices	Concentration
Allantoin	x			N/A
Alginate and derivatives			x	N/A
Aloe vera			x	N/A
Birch bark extract		x		100 mg/g
Bisabolol	x			N/A
Caprylic/Capric triglycerides	x			N/A
*C. asiatica* and pure compounds	x	x		N/A
Essential fatty acids			x	N/A
Honey			x	N/A
Metal salts and oxides	x	x		Zinc oxide: 150–500 mg/g ^1^
Proteolytic enzymes		x		Proteolytic enzymes of *Ananas comosus* (L.) Merr.: 2–5 g/22 g ^1^ Collagenase: 0,6 U/g ^1^
Petrolatum derivatives			x	N/A
Salicylic acid		x		5 mg/g ^1^
Silicones			x	N/A
Squalene or squalane	x			N/A
Sunflower oil	x			N/A
*T. vulgare*		x		150 mg/g ^1^
Trolamine		x	x	6.7 mg/g ^1^
Vitamin A and derivative		x		212.5 U.I./g ^1^
Vitamin B5 and/or derivatives	x	x	x	Dexpanthenol: 50 mg/g ^1^
Vitamin D3		x		21.25 U.I./g ^1^
Vitamin E and derivative	x		x	N/A

Only the ingredients responsible for skin repair activity in each category are mentioned. ^1^ Active substances concentration of topical medicines, available in Medicinal Product Details section in Informed. N/A: the concentration of the ingredient is not available.

**Table 2:** Summary of the mechanisms of action of the top 3 most used ingredients analyzed.

Ingredients	Wound Healing Phase Targeted	Skin Repair Mechanism	References
Metal salts and oxides	Hemostasis phase Inflammatory phase Proliferation phase Remodeling phase	Antioxidant effect Neutralization of ROS and other peroxides; Antimicrobial and biocidal effect Restoring dermal matrix ↑ Breakdown of collagen fragments promoting wound debris; ↑ Temperature; ↑ Oxygen tension; ↑ Fibroblasts proliferation; Hemostatic effect ↑ Blood absorption and platelet aggregation; Anti-inflammatory effect Antinociceptive effect Blocks *N*-methyl-D-aspartate receptors; Angiogenic effect	[31,32,35,36,37,39,40,41,46,47,49,51,52,58,59,60,61,62,63,64,65,66,67,68,69,70]
Silicones	Hemostasis phase Proliferation phase Remodeling phase	Hemostatic effect: Promotes blood absorption and platelet aggregation. Restoring dermal matrix ↑ Adhesion and fibroblast proliferation; ↑ Temperature; ↑ Oxygen tension. Occlusive effect ↑ Skin hydration; Acts as barrier for bacteria.	[81,84,86,87,88,89,90,91,92,93,94]
Petrolatum derivatives	Inflammatory phase Proliferation phase Remodeling phase	Occlusive effect ↑ Skin hydration; ↑ Collagen synthesis; ↑ Angiogenesis; ↑ Dead tissue and fibrin breakdown; ↓ Scab formation; Act as barrier for bacteria.	[96,98,99,100,101,103,104,105,106,107,108,109,110,111,112,113,114,115]
Vitamin E and derivatives	Inflammatory phase Remodeling phase	Antioxidant effect Neutralization of ROS and RNS; Anti-inflammatory effect: ↓PGE2 levels; inhibit iNOS mRNA expression, NO production and COX-2 activity; restoring dermal matrix: ↑ Collagen synthesis ↓ Collagen degradation	[20,117,124,125,126,127,128,129,130,131,132]
Dexpanthenol	Proliferation phase	Moisturizing effect: ↑ Retention of water; Restoring dermal matrix: ↑ Fibroblast proliferation Restoring barrier: ↑ Molecular mobility of several lipid and protein segments of the SC ↑ Synthesis of coenzyme A ↑ Fatty acid and sphingolipid synthesis	[135,136,138,139,140,141,142,143,144,145,146]
Vitamin A and derivatives	Inflammatory phase Proliferation phase Remodeling phase	Anti-inflammatory effect: ↓ NO production; Restoring dermal matrix: ↑ Collagen synthesis ↓ Collagen degradation ↑ Fibroblast proliferation Inhibition the action of metalloproteinases; Promotes endothelial cell migration;	[153,163]
Alginate and derivatives	Hemostasis phase Inflammatory phase	Antioxidant effect Anti-inflammatory effect Antimicrobial effect Moisturizing effect Exudate absorption	[181,184,185]
*C. asiatica* and pure compounds	Inflammatory phase Proliferation phase Remodeling phase	Antioxidant effect: Anti-inflammatory effect: ↑ Angiogenesis Restoring dermal matrix: ↑ Collagen synthesis ↑ Fibroblast proliferation	[166,170,171,172,173,174,175,176,177,178,179]

Footnotes: ↑ increase; ↓ reduction.

## Data Availability

Data is contained within the article.
